# Performance Analysis and Numerical Evaluation of Mixing in 3-D T-Shape Passive Micromixers

**DOI:** 10.3390/mi9050210

**Published:** 2018-04-28

**Authors:** Mahmut Burak Okuducu, Mustafa M. Aral

**Affiliations:** 1 School of Civil and Environmental Engineering, Georgia Institute of Technology, Atlanta, GA 30332, USA; mbokuducu@gatech.edu; 2 Department of Civil Engineering, Bartin University, Bartin 74100, Turkey

**Keywords:** micromixers, microfluidics, CFD, grid type, finite volume method, finite element method, numerical diffusion, artificial diffusion, false diffusion

## Abstract

In micromixer devices, laminar characteristics of the flow domain and small diffusion constants of the fluid samples that are mixed characterize the mixing process. The advection dominant flow and transport processes that develop in these devices not only create significant challenges for numerical solution of the problem, but they are also the source of numerical errors which may lead to confusing performance evaluations that are reported in the literature. In this study, the finite volume method (FVM) and finite element method (FEM) are used to characterize these errors and critical issues in numerical performance evaluations are highlighted. In this study, we used numerical methods to evaluate the mixing characteristics of a typical T-shape passive micromixer for several flow and transport parameters using both FEM and FVM, although the numerical procedures described are also equally applicable to other geometric designs as well. The outcome of the study shows that the type of stabilization technique used in FEM is very important and should be documented and reported. Otherwise, erroneous mixing performance may be reported since the added artificial diffusion may significantly affect the mixing performance in the device. Similarly, when FVM methods are used, numerical diffusion errors may become important for certain unstructured discretization techniques that are used in the idealization of the solution domain. This point needs to be also analyzed and reported when FVM is used in performance evaluation of micromixer devices. The focus of this study is not on improving the mixing performance of micromixers. Instead, we highlight the bench scale characteristics of the solutions and the mixing evaluation procedures used when FVM and FEM are employed.

## 1. Introduction

During the past two decades, numerous experimental and numerical modeling studies have been performed for the design of microfluidic devices in which various geometric designs are recommended to improve the mixing efficiency in these devices. Due to widespread use of microfluidic devices in diverse disciplines it is obvious that these studies will grow exponentially in the future with the main goal of improving the mixing performance. Micromixers are essential components of microfluidic systems [1] in which fluid mixing is developed in miniaturized devices. These devices are extensively used in chemical, biological, medical, and environmental applications [2,3]. Fast and thorough mixing of two or more samples at micro scales are the purpose of micromixers. Major benefits of microfluidic devices, as opposed to their macro counterparts, are their high surface-to-volume ratios, small amount of sample requirement for analysis, low cost, short time operating conditions, high efficiency, compactness of the device, and safety in case of hazardous chemical usage [2,4]. Micromixers are usually categorized as active and passive devices depending on the presence of external disturbance effects which are employed to enhance mixing efficiency in active micromixers [2,5,6,7]. Due to important advantages of passive micromixers [8,9], passive micromixers have been widely used and investigated by researchers in which improved flow, transport and mixing behavior of these devices are studied.

In macro-scale systems, mixing mainly develops due to turbulent flow characteristics by which liquids are naturally stirred and the impact of molecular diffusivity in turbulent flow regimes can be ignored since turbulent diffusivity is the dominant mixing parameter [10]. Mixing at micro-scales is a more challenging process due to strictly laminar flow regimes that are observed in these devices where mixing is performed at very low molecular diffusivities (e.g., *D* = 10^−9^–10^−11^ m^2^/s [11]) for many chemical and biological solutions. The advection dominant transport regimes (i.e., high Peclet (Pe) number) that develop in these devices lead to problems in numerical solution of these problems.

Computational Fluid Dynamics (CFD) simulations are usually employed to investigate fluid flow and solute transport at micro scales. The finite volume method (FVM) and finite element method (FEM) are the two numerical techniques that are commonly used in these applications. Complications in numerical solution often arise depending on the characteristics of the numerical method employed along with other factors such as discretization techniques, grid size and properties, and boundary conditions used. In aggregate, these choices may significantly affect the reliability of the numerical performance evaluation results. The two-important numerical complication in CFD studies are the numerical diffusion (or false diffusion) and numerical dispersion effects. These effects may prevent the proper interpretation of the results of fluid flow and mixing analysis performed. Numerical diffusion arises from the numerical approximation of the advection term in the flow equation [12,13,14]. On the other hand, lack of dispersion yields numerical instability problems (i.e., oscillations) in the solution because of high gradients of the variables that appear in the flow and transport domain. These oscillations need to be eliminated as much as possible to obtain reliable numerical solutions. The degree of numerical errors in these solutions are associated with the discretization schemes that are used in the solution of the governing equations (i.e., the magnitude of truncation errors) as well as other numerical and physical parameters used, such as grid size, grid type, and diffusivity (or viscosity for fluid flow). Godunov [15] showed that a linear monotone scheme which does not create over- and undershoots can be at most first-order accurate. However, higher order schemes are more commonly preferred in applications due to their lower numerical diffusion effects as it is preferred in this study as well. In CFD applications, especially for advection dominant problems, FVM seems to be more advantageous and may provide relatively more consistent results [16] because of its conservative mass, momentum, and energy formulation structure. It is also shown that if the flow direction is orthogonal to the grid lines or in other words, the flow direction and grid lines are orthogonally aligned in the computational domain, the FVM does not produce high false diffusion effects [17]. The amount of false diffusion created increases when the angle between streamlines and gridlines approaches to 45°. In most CFD applications, maintaining mesh and flow alignment in a computational domain in this sense is not practical and numerical solution inevitably exhibits some effect of false diffusion which needs to be accounted for in mixing performance evaluation. Meanwhile, FEM suffers from stability as well as numerical diffusion problems, especially when working with advection dominant systems [18]. In this application, although it is possible to avoid unwanted node-to-node oscillations by grid refinements, this approach is not practical because of high cost of numerical simulations at fine grids that are necessary. A practical approach to stabilize oscillations in FEM is known as artificial diffusion (or artificial viscosity for fluid flow) [17,19] in which diffusion constant (or viscosity) is artificially increased to eliminate the instability in the numerical solution at the cost of excess diffusion that is added to the system. This approach may affect the interpretation of the outcome of the mixing performance of micromixers since the excess diffusion changes the physics of the problem and for such cases it is not clear if mixing outcome is artificial due to the added artificial diffusion and false diffusion effects or real due to the molecular diffusion of the fluids that are mixed. Researchers have introduced several techniques for both FVM and FEM to solve fluid flow and mass transport equations more accurately by suppressing the negative effects of false diffusion and artificial diffusion in CFD applications. Unfortunately, none of these methods are problem-free. The most popular techniques used in stabilization of FEM and high-resolution schemes applied in FVM can be found in [14,20], respectively, with evidence of deficiencies in both of these techniques.

Significant effort is necessary in reporting the outcome of the mixing performance in numerical studies. As stated earlier, in micromixers molecular diffusion effect is typically the only mixing mechanism. The mixing effects of diffusion may be acutely masked by the aforementioned numerical errors. Since complete elimination of numerical errors is not possible, quantifying the presence of these errors are necessary to provide reliable and unsusceptible results in the evaluation of mixing performance of micromixers.

In investigating several micromixer studies, Liu [13] extensively discussed the extent of numerical diffusion problems in micromixer literature. He also stated that numerical diffusion effects of high-order schemes were rarely discussed in the literature, with which we agree. In his study, Liu conducted several tests using different numerical schemes under various grid size, flow, and transport conditions for a three-dimensional microchannel mixer. He utilized hexahedral mesh type in FVM and proposed a set of equations to quantify average numerical diffusion which results from both flow and scalar transport solutions. He also studied tetrahedral mesh type and advised that the use of this type of a mesh should be avoided especially for scalar transport solution since the amount of false diffusion and computational cost is higher than that of hexahedral mesh type. By examining various higher order schemes (e.g., Second-Order Upwind, QUICK [21], MUSCL [22]), he found that false diffusion can be significantly reduced if higher order schemes are used instead of first order upwind schemes.

Bailey [12] investigated false diffusion effects using FVM in a two-dimensional test problem for both structured and unstructured grids applying first and second-order upwind methods and quantified the amount of false diffusion that is generated. He also developed a set of procedures to estimate and manage the required grid size by which the false diffusion amount can be reduced in steady micro scale mixing simulations.

Soleymani et al. [23] investigated flow dynamics and mixing in a T-shape micromixer using FVM and discussed several approaches to study and diminish numerical diffusion effects at moderate cell Pe numbers. They performed spatial discretization using high-order QUICK scheme, locally refined the mesh, and increased the diffusion constant artificially to overcome high computation cost. In their study they reported that numerical simulation results were used for optimization purposes rather than the physical mixing evaluation of the micromixer.

On the other hand, in several micromixer studies [8,24,25,26,27,28] where FEM was employed, the authors did not report the instability and false diffusion effects although they have studied advection dominant systems. In these applications, depending on the stabilization method applied, mixing performance results reported may show significant variations depending on the degree of numerical treatment applied.

In view of these arguments, the purpose of this study is to systematically investigate, quantify, and show the numerical diffusion effects in micromixer CFD simulations. For this purpose, various fluid flow, transport, and idealizations (grid configurations) were utilized when both FVM and FEM is used in solution. Quantification and recommendation of application ranges of these techniques are provided for T-shape mixers for future reference. However, the analytical methods used in the evaluation are generic and applicable to other mixer devices as well. Although both FVM and FEM is used in this study, the main emphasis is on FVM due to its extensive usage in micromixer applications and widespread availability of this method in several commercial and non-commercial CFD packages.

## 2. Mathematical Model

In micro channels, fluid flow and transport equations are defined using a set of partial differential equations which are derived from the well-known mass, momentum, and energy conservation principles. Here we will assume that mixing fluids are of constant density, constant viscosity, miscible, and non-reactive with identical physical properties. Gravitational and temperature effects were not considered in the mathematical model due to their negligible effects in micromixer applications. The flow regime is assumed to be laminar and the fluid is incompressible. Based on these assumptions, the governing equations which describe the flow field and mass transport are the continuity equation, Equation (1); Navier–Stokes equation, Equation (2); and the advection-diffusion (AD) equation, Equation (3).

(1) ∇⋅u=0

(2) $$\rho \left[\frac{\partial u}{\partial t}+u\cdot \nabla u\right]=-\nabla p+\mu \nabla^{2}u$$

(3) $$\frac{\partial c}{\partial t}+u\cdot \nabla c=D\nabla^{2}c$$

Equations (1) and (2) represent mass and momentum conservation respectively, where, ρ is the density of fluid (kg/m^3^), µ is the dynamic viscosity of the fluid (Pa·s), **u** is the velocity vector (m/s), *p* is the pressure (Pa). For all scenarios, the stationary velocity field obtained from the solution of Equations (1) and (2) is used to simulate the steady-state transport of solute using Equation (3) in which *c* is the concentration of the solute (mol/m^3^) and *D* is the molecular diffusion coefficient (m^2^/s). Throughout this study, the molecular diffusion coefficient of the transported solute is chosen as *D* = 3 × 10^−10^ m^2^/s which corresponds to crystal violet dye [29].

**Figure 1:**
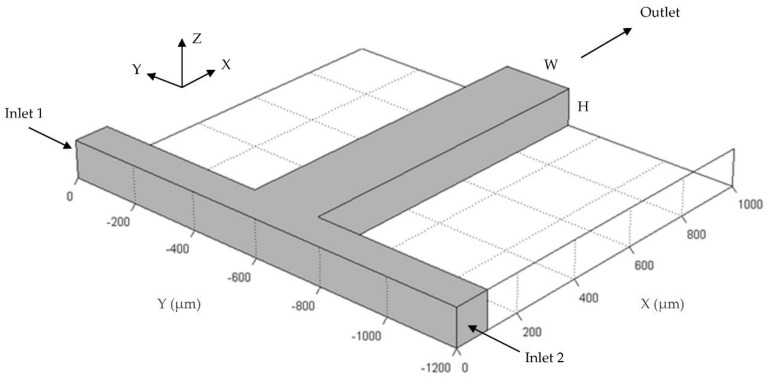
Three-dimensional view of T-shape passive micromixer.

In this study, flow and mixing analysis of two liquids were investigated in a typical three-dimensional (3-D) T-shape passive micromixer which consists of two equal length inlet channels and a mixing channel as shown in Figure 1. Although the analytical techniques used for mixing evaluation is applicable to other micromixer geometries, in this study our focus will be on T-mixers. Inlet channel lengths were chosen as 500 µm with a square cross section of 100 µm × 100 µm considering that flow will be fully developed before entering the mixing channel for the highest Reynolds number (Re) scenario tested in this study (i.e., Re = 100). The mixing channel length is 1000 µm with a width (*W*) and height (*H*) of 200 µm and 100 µm respectively. The T-shape passive micromixer dimensions are consistent with the T-shape passive micromixers studied in the literature [25,30,31].

Physical properties of mixing fluids and appropriate boundary conditions used in simulations are given in Table 1.

In micromixer studies, flow and transport characteristics can be defined through two dimensionless numbers, i.e., the Reynolds (Re) number and Peclet (Pe) number, as given in Equation (4), respectively,

(4) Re = U¯Dhν; Pe = U¯DhD

where, $\bar{U}$ is the average flow velocity (m/s), *v* is the kinematic viscosity (m^2^/s), *D_h_* is the characteristic length which is assumed to be hydraulic diameter of a rectangular duct (m) and defined as, *D_h_* = 4*A*/*P* in which *A* is the area and *P* is the wetted perimeter of the duct.

Typically, Re = 2300 is considered as the critical point [2,32] after which flow regime starts to change from laminar flow to turbulent flow. In microfluidic applications, Re number is generally far below than this transition point (usually Re < 100 [3]). Therefore, the flow regime in micro channels can be evaluated as strictly laminar. High Peclet number indicates that mass transport is due to advection rather than diffusion. In this study, Re and Pe numbers were calculated in the mixing channel of T-mixer. In addition to these characteristic numbers, the cell Peclet number (Pe_Δ_ = *U*Δ*x*/*D*) and cell Reynolds number (Re_Δ_ = *U*Δ*x*/*v*) are used to observe the accuracy and stability of the numerical solution. For example, in case of scalar transport, when cell Peclet number is greater than 1, FEM yields oscillatory solutions since large concentration gradients could not be adequately captured. Similarly, several second or higher-order numerical schemes used in FVM (e.g., central difference, second-order upwind etc.), show numerical instability when cell Peclet number is greater than 1. In this analysis, cell Peclet and cell Reynolds numbers were calculated as an average value since the velocity magnitude is a local variable in the computational domain.

The mixing performance of simulations were quantified using mixing index (MI) as shown in Equation (5) [32,33] in which σ is the standard deviation of the concentration in a given cross-section, *c_i_* and *c_m_* are concentrations at *i*th sample point and mean concentration on the cross-section respectively, and *N* is the total number of sample points.

(5) $$\mathrm{MI}=1-\sigma ;\;\sigma =\sqrt{\frac{1}{N}\sum_{i=1}^{N}\left(\frac{c_{i}-c_{m}}{c_{m}}\right)}$$

Mixing efficiency changes between 0 and 1 corresponding to unmixed (i.e., 0% mixing) and completely mixed (i.e., 100% mixing) states, respectively. An acceptable mixing efficiency in micromixer applications is usually higher than 80% [32].

The average numerical diffusion in the solution domain can be quantified using the procedure proposed in [13]. In this approach, effective diffusivity in a numerical solution is defined as the sum of the physical diffusivity in the system and the numerical diffusion as given in Equation (6). Here, *D_M_* is molecular diffusivity of the mixing fluid and *D_N_* is numerical or false diffusivity.

(6) $$D_{Effective}\approx D_{M}+D_{N}$$

The effective diffusivity given in Equation (6) can be computed using the results of numerical solution of scalar transport equation as formulated in Equation (7).

(7) $$D_{Effective}=\frac{c_{inlet}^{2}-c_{outlet}^{2}}{2\tau {\left(\nabla c\right)}_{\forall }^{2}}$$

where,

(8) $$c_{inlet}^{2}=\frac{1}{Q}\int_{A}n\cdot uc^{2}dA$$

(9) $$c_{outlet}^{2}=\frac{1}{Q}\int_{A}n\cdot uc^{2}dA$$

(10) $$\nabla c_{\forall }^{2}=\frac{1}{\forall }\int_{\forall }\left(\nabla c)\cdot (\nabla c\right)d\forall$$

In these equations, *c* is concentration, **n** is unit vector, ∀ is the volume of the micromixer, *Q* is the volumetric flow rate, $c_{inlet}^{2}$ and $c_{outlet}^{2}$ are flow rate weighted average value of concentration at the inlet and outlet respectively, $\nabla c_{\forall }^{2}$ is the volume average, *τ* is the mean residence time of the flow, τ=∀/Q for unobstructed T-shape mixers. For obstructed micromixers, actual residence time of solute particles must be used in Equation (7). Given Equation (7), numerical diffusivity can be calculated by setting the molecular diffusion constant to zero and the effective diffusivity calculated would give the average value of numerical diffusion expected in the solution of the scalar transport equation. Therefore, in this study, two simulations were performed for each scalar transport scenario. In the first simulation, molecular diffusion constant was set to zero and the AD equation was solved and from the numerical solution the effective diffusivity was calculated using Equation (7) which in this case would equal to the estimated numerical diffusion in the solution (*D_Effective_ ≈ D_N_*). The second simulation was conducted using the molecular diffusion constant of *D_M_* = 3 × 10^−10^ m^2^/s. For this case the effective diffusivity calculated in Equation (6) will now include the effects of both the molecular diffusion and numerical diffusion as described in Equation (6) (*D_Effective_ ≈ D_M_ + D_N_*). The analysis of the results of these two solutions will be discussed in the following sections.

In this study, numerical solution of the governing partial differential equations was carried out using both FVM and FEM with two different CFD tools. These are OpenFOAM (v5.0, OpenFOAM Foundation, OpenCFD Ltd., Bracknell, UK) and COMSOL Multiphysics (v5.3a, COMSOL AB, Stockholm, Sweden). OpenFOAM is a non-commercial and open source CFD software based on FVM application. In FVM both structured and unstructured meshes can be utilized. To solve the governing equations for steady-state, incompressible, and laminar flow *simpleFOAM* solver was used in which the SIMPLEC (semi-implicit method for pressure linked equations-consistent) [34] algorithm was used for solving pressure-velocity coupling. Advection and diffusion terms in the flow equation were discretized using second-order upwind scheme (i.e., Gauss linearUpwind) and second-order central difference scheme (i.e., Gauss linear) respectively. The AD equation was solved utilizing the *scalarTransportFoam* solver in which advection terms were discretized using first-order upwind and multiple second-order accurate numerical schemes built-in OpenFOAM (e.g., second-order upwind [35], QUICK, MUSCL, *limitedLinear* [36]) to observe and ensure the boundedness of concentrations for both structured and unstructured meshes used in this study. Diffusion terms in AD equation was treated using second-order central difference scheme. In all simulations, iterations were continued until final residuals of flow and transport equations fall below 1 × 10^−12^ which was assumed to yield converged solutions. The structured and unstructured meshes used in OpenFOAM simulations were generated using Gmsh [37].

COMSOL Multiphysics, which is a FEM based commercial CFD package, was employed to analyze the artificial diffusion effects. Fluid flow and scalar transport were simulated using the laminar flow and transport of diluted species tools and computational mesh was generated using the geometry interface in the software. For uniformity, discretization accuracy, and convergence levels of flow and transport equations were set equivalent to that of OpenFOAM simulations which was determined from grid convergence analysis. To dampen the oscillation effects in the solution of Navier–Stokes and AD equations, COMSOL provides two stabilization options which are referred to as consistent and inconsistent methods. In the consistent method, directions and gradients of variables are evaluated by the solver and appropriate corrections are made in the regions where stabilization is required. In inconsistent stabilization, physical diffusivity (mass or molecular) is artificially increased with a tuning parameter to obtain a stable solution. In this analysis, simulations were conducted for both correction methods.

FEM and FVM simulations conducted in this study were performed on a personal computer with an Intel Core i7-6900K processor which was overclocked to run at 4.2 GHz (Intel Corporation, Santa Clara, CA, USA) and 64 GB 3200 MHz random-access memory (RAM).

## 3. Grid Independence Analysis

**Figure 2:**
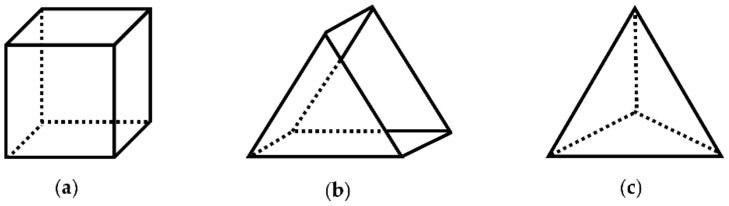
Three-dimensional mesh element types: (**a**) hexahedron; (**b**) prism; (**c**) tetrahedron.

The numerical error generated in 3-D micromixer analysis is a function of grid size, local velocity magnitude, and orientation of velocity vectors with respect to the grid boundaries which may be identified as grid alignment effects [13]. Grid element type chosen becomes more important in FVM since the non-orthogonal alignment of mesh boundaries and flow direction will increase the effect of false diffusion in the solution since in FVM continuity of the gradients of the unknown parameters across boundaries are used to develop the governing matrix equations. Considering these factors several test cases were designed in this study to observe and quantify the effects of numerical errors. Micromixer simulations were designed for five different flow scenarios (i.e., Re = 0.1, 1, 10, 50, 100) and for three type of mesh structures (i.e., structured hexahedral, structured prism, and unstructured tetrahedral), which are generated using the elements shown in Figure 2. Designed test cases with simulation parameters are given in Table 2.

**Figure 3:**
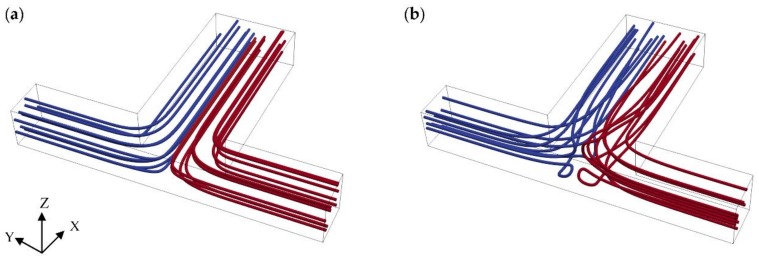
Flow profile at the beginning of mixing channel (Figures show the T-mixer region between *x* = 0 and 500 µm and *y* = −250 and −700 µm): (**a**) separated (or stratified) flow at Re = 0.1; (**b**) periodic (or Vortex) flow at Re = 100.

**Figure 4:**
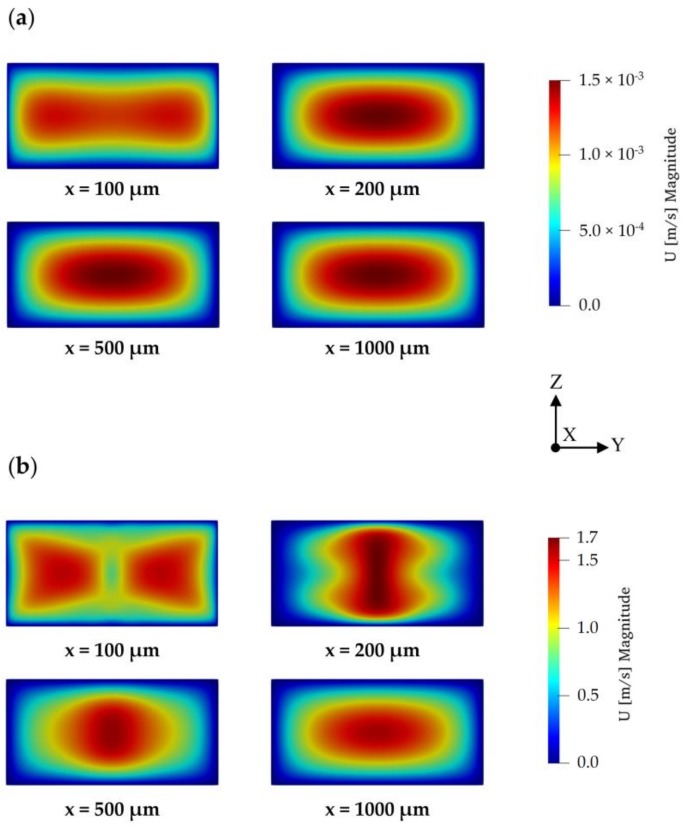
Velocity profile at four cross-sections in the mixing channel of the T-mixer (Color ranges were set to that of *x* = 200 µm plane): (**a**) Re = 0.1; (**b**) Re = 100.

In the T-mixer, the fluid flow solutions showed that if the flow profile in the inlet channels are not fully developed before entering the mixing channel, stratified (or separated) flow regions occur at the beginning of the mixing section implying that the liquids which approach to the mixing channel from two different inlet streams travel side-by-side along the mixing channel without rotation in the *z*-direction for all Re scenarios. If, however, the flow in the inlet channels are fully developed, periodic (or vortex) flow type may be observed at the entrance section of the mixing channel for Re = 50 and 100 scenarios. Therefore, inlet channel lengths of the T-mixer were selected to be long enough to create a fully developed flow profile for high Re number scenarios studied which is 500 µm. The flow regimes observed in the T-mixer are shown in Figure 3 for Re = 0.1 and 100 cases. In addition, velocity profiles at different cross-sections in the mixing channel are shown in Figure 4. As shown in Figure 4, the most non-uniform flow in the T-mixer occurs at *x* = 200 µm in the mixing channel at Re = 100. Investigation of the fully developed flow effects in the inlet channels on mixing performance is beyond the scope of this study and this point will not be further discussed here. We note that fluid flow results reported in this study agree with the findings of extensive studies reported in the literature on this subject [23,29].

In CFD applications, grid generation is a pivotal stage since spatial discretization errors will inherently affect the numerical solution. Theoretically, it is known that temporal and spatial discretization errors asymptotically approach zero by reducing the time step and mesh element size; however, as the mesh is refined, computational cost and the solution time increases in parallel. Therefore, an optimized solution in terms of accuracy, computational cost, and solution time becomes important. The aim of systematic grid convergence studies in CFD applications is to estimate the amount of spatial discretization error between different mesh densities and determine a practical mesh level at which computational cost is feasible and spatial error is controlled [38]. Grid Convergence Index (GCI) analysis is a well-established and commonly used method for systematic grid convergence studies as described in [39,40,41]. In this study, GCI method was used to determine the order of convergence of the simulations and verify that solution is independent of mesh resolution. GCI study was made using FVM for three different mesh structures (i.e., structured hexahedral, structured prism, and unstructured tetrahedral) separately for the highest Reynolds number scenario (i.e., Re = 100) since the most complex flow regime was observed at this Re number as can be seen in Figure 3. For hexahedral and prism element types, four different grid levels (i.e., L_1_, L_2_, L_3_, L_4_) were created with a constant refinement ratio of 1.5 in *x*, *y*, and *z* coordinate directions. For the tetrahedral grid type, total element number was fixed around the mesh density of prism type as given in Table 3.

**Figure 5:**
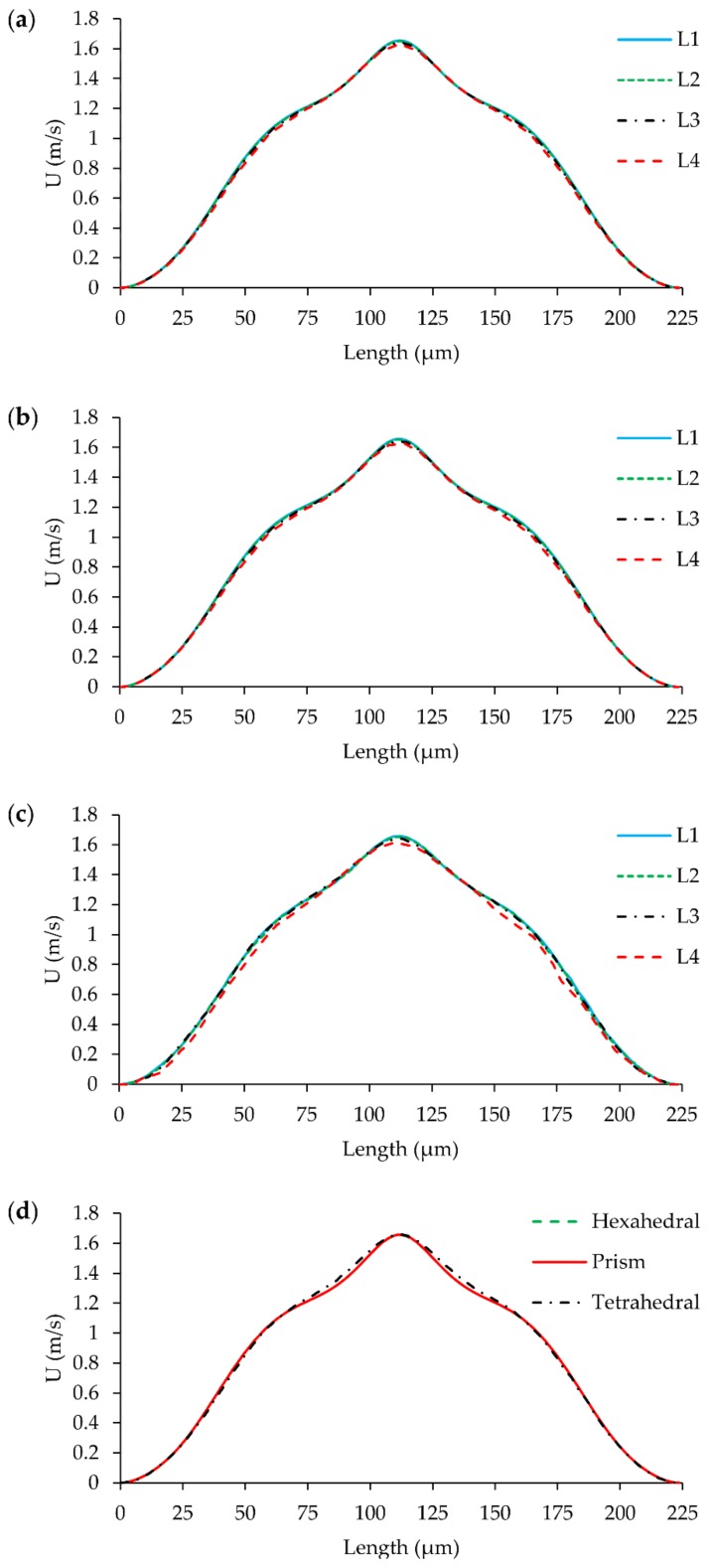
Velocity distribution on the plane at *x* = 200 µm from L_1_, L_2_, L_3_, and L_4_ grid level simulations: (**a**) hexahedral; (**b**) prism; (**c**) tetrahedral; (**d**) hexahedral vs. prism vs. tetrahedral solutions at L_1_.

**Figure 6:**
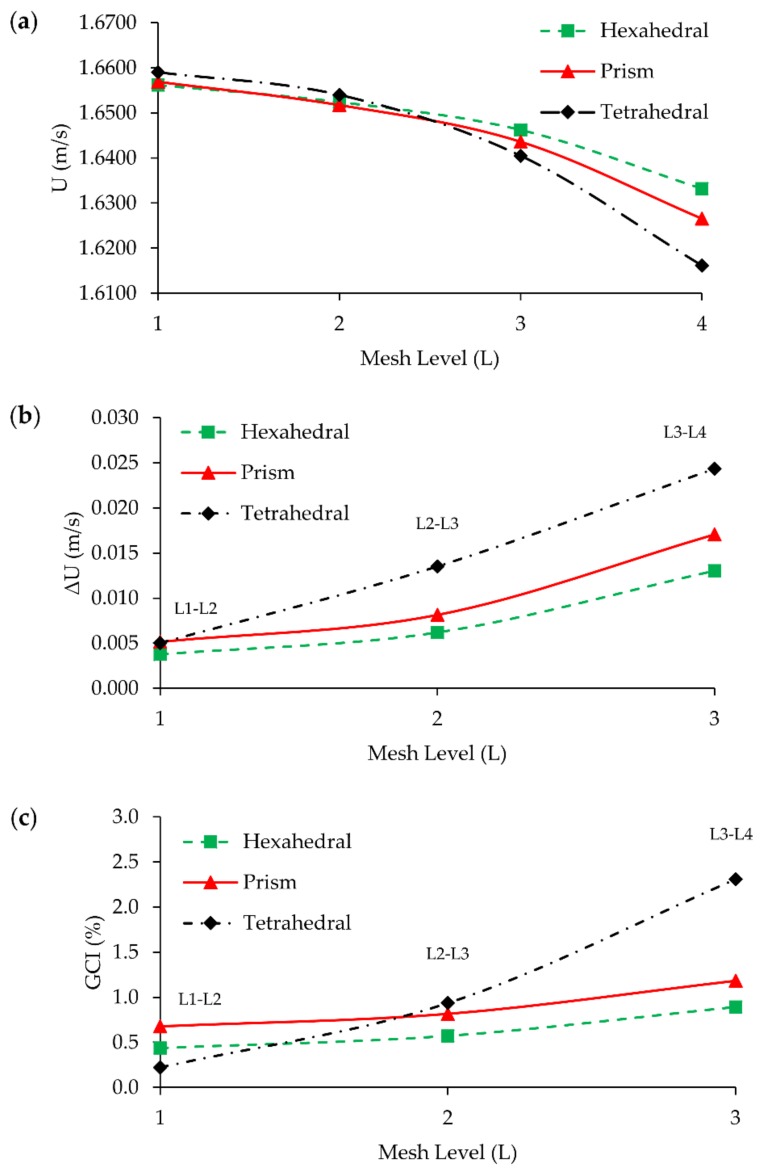
Grid convergence results: (**a**) maximum velocity magnitude on the plane at *x* = 200 µm obtained from L_1_, L_2_, L_3_, and L_4_ grid levels for all mesh types; (**b**) maximum velocity magnitude difference between mesh levels; (**c**) GCI between mesh levels.

GCI study was performed using the flow solution in the T-mixer. Maximum velocity at *x* = 200 µm plane in the mixing channel was selected as GCI parameter since the most complex flow profile was observed at this point as can be seen in Figure 4b. Crosswise velocity distributions on this plane for three different mesh structures at different mesh densities are shown in Figure 5. It can be seen in Figure 5 and Figure 6, that the GCI study results show that flow field at Re = 100 was resolved consistently for all mesh types using second-order discretization schemes for advection and diffusion terms in Equation (2). Computed velocity values, which are used in GCI study, are approaching to a constant point asymptotically when meshes are refined as plotted in Figure 6a. This asymptotic level can be considered to be the exact solution of flow equations. Similarly, the difference in computed velocities and GCI of two consecutive mesh levels are reduced with grid refinement, as shown in Figure 6b,c.

As given in Table 2, the minimum and maximum average cell Re number scenario (at Re = 100) are 1.50 and 4.95, respectively, for hexahedral mesh. These moderate numbers indicate low advection dominance in the system. In other words, advection and diffusion terms in Equation (2) feed the numerical solution almost equally which reduce numerical instability and false diffusion in the system. This is actually because of the high kinematic viscosity of the fluid. Results showed that, although mesh and flow alignment is not maintained in prism and tetrahedral meshes, the solution was not affected by numerical errors significantly due to resulting moderate cell Re numbers (assuming cell Re number in prism and tetrahedral domain is close to that of hexahedral).

**Figure 7:**
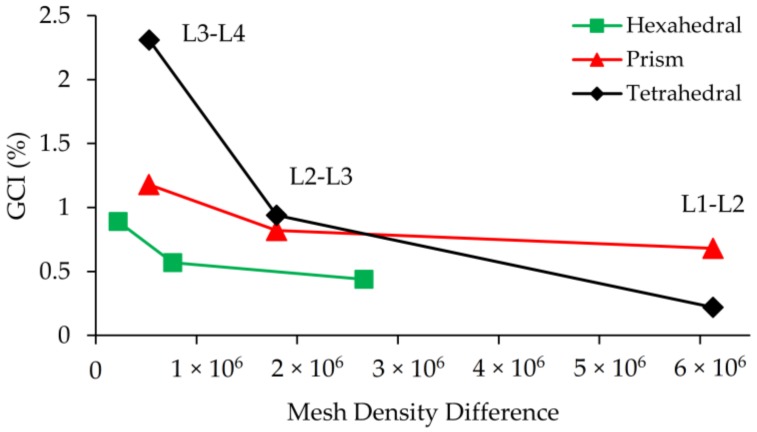
GCI vs. mesh density difference between grid levels.

While the velocity profile on the plane at *x* = 200 µm could be successfully resolved for all mesh types at L_1_, as shown in Figure 5d, prism and tetrahedral mesh density is about 2.3 times more than the hexahedral mesh type which is significant in terms of computational cost. The maximum difference in solutions was observed to be 2.31% between L_4_ and L_3_ of tetrahedral mesh with a mesh density difference of roughly 5 × 10^5^. Also, for this type of idealization, while the difference between solutions at L_2_ and L_1_ is only 0.22%, L_1_ simulations were performed using 6 million more elements. GCI values and mesh density differences between mesh levels are shown in Figure 7.

As it can be seen in Figure 7, sharp changes in the slopes of these lines show that accuracy gain is higher than grid number growth when refinement is maintained between L_4_ and L_2_ for all grid types. However, after the L_2_-L_3_ point, mesh density increases excessively with respect to GCI, implying that further grid refinement does not contribute to numerical accuracy significantly, but it increases the computational cost unreasonably. If the 1% GCI difference is determined as the limit, simulations can be done at L_2_ for all mesh types, but in this study L_1_ mesh density was employed for all test cases and mesh types to investigate the magnitude of numerical diffusion errors in scalar transport solution more accurately. The reason for selection the finest grid for Re = 0.1, 1, 10, 50 scenarios is that each case provides a different average cell Pe number by which the amount of numerical diffusion can be observed. It is clear that increasing the grid size will also increase average cell Pe number. Thus, instead of conducting additional simulations for different grid sizes, all simulations were conducted at the finest grid size and the amount of numerical diffusion was evaluated for various average cell Pe numbers which range between 5 and 5000 for Re = 0.1 and 100 scenarios respectively. These results are provided in Table 2.

**Figure 8:**
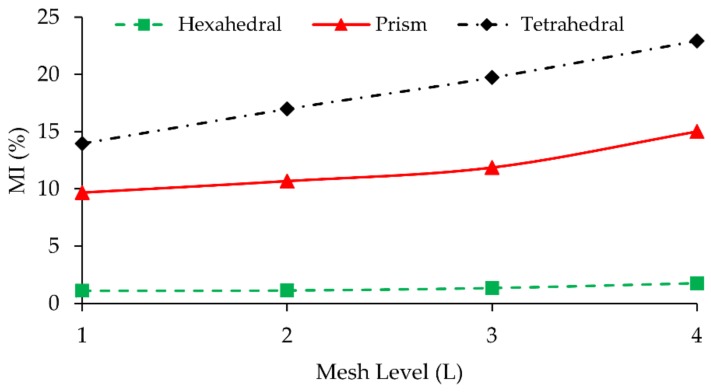
Outlet mixing index of hexahedral, prism, and tetrahedral mesh types at L_1_, L_2_, L_3_, and L_4_.

In the literature, the mixing efficiency parameter is also used in grid refinement analysis. When outlet mixing efficiency is used in grid convergence analysis [9,27,42,43], important discrepancies are observed in grid convergence analysis, especially for prism and tetrahedral mesh types. As it is observed in this study, this discrepancy was also mentioned in [13]. Although mesh refinement reduced numerical errors in the scalar transport solution as shown in Figure 8, mixing efficiencies obtained significantly differ from one another for different mesh types. This is due to numerical errors and numerical diffusion generated during the solution of transport equation which significantly effects the mixing estimates obtained. While hexahedral mesh type predicts outlet mixing efficiency with a 2.33% difference between L_2_ and L_1_, this amount sharply increases to a value around 9% and 14% at same mesh levels for prism and tetrahedral mesh types, respectively, indicating higher numerical errors and thus higher mixing efficiency.

**Figure 9:**
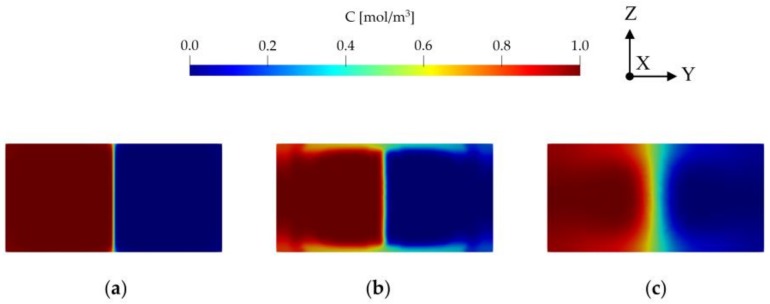
Concentration distribution at the outlet of T-mixer for Re = 100 scenario at L_1_: (**a**) hexahedral; (**b**) prism; (**c**) tetrahedral.

As shown in Figure 9, there is a significant divergence between outlet concentration distributions of different mesh types even at L_1_. It needs to be pointed out that use of the mixing efficiency parameter for grid convergence studies may not be appropriate unless cell Pe number is quite low. At high cell Pe numbers, several factors may affect the solution in addition to the grid size and grid type, such as numerical diffusion, oscillations, characteristic of numerical scheme employed, and mesh type etc. In this grid study, the average cell Pe numbers are in the range of 5000 at L_1_ and 16,500 at L_4_ for the hexahedral mesh type which are significantly high values which require special discretization techniques. This in turn affects the mixing efficiency estimated at the outcome. Even when mesh and flow alignment were maintained in the hexahedral domain, there was a significant difference between mixing index values at the different mesh levels. For moderate cell Pe numbers this problem may be tolerable. However, for high cell Pe number scenarios, the numerical results may imbed important effects of numerical diffusion and thus erroneous mixing efficiency values.

**Figure 10:**
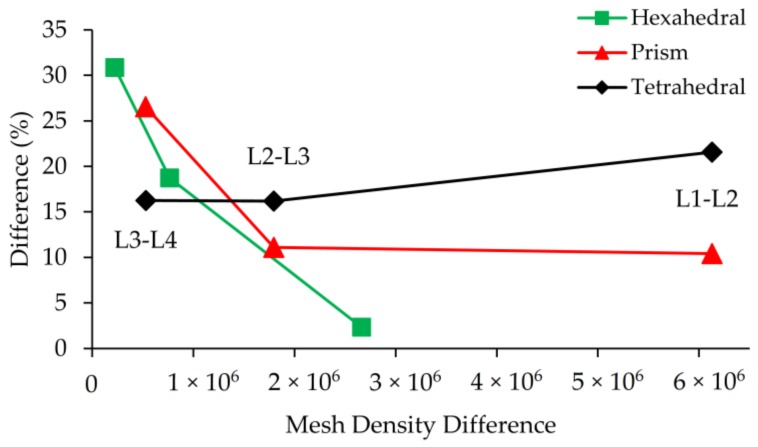
Error (%) between mesh levels in terms of outlet mixing efficiency parameter vs. mesh density difference between grid levels.

The comparison of Figure 7 and Figure 10 shows a significant inconsistency when flow and scalar transport results are considered as grid study parameters. For instance, at Re = 100, scenario difference between L_4_ and L_3_ was observed as 2.3% for the tetrahedral type when flow field solution is used in GCI, yet this difference increased to more than 20% when the grid study was conducted with a scalar transport solution and mixing efficiency. Even in the best-case scenario, which is the hexahedral mesh solution, there is a considerable disagreement between the results when two grid convergence parameters are compared. The GCI value between L_2_ and L_1_ is 0.44% when the maximum velocity magnitude is employed in the grid study, whereas, the difference between the same levels increases to 2.33% if outlet mixing efficiency is used in convergence analysis. For other mesh types, this situation is much worse, namely while prism mesh shows around 10% difference between L_2_ and L_1_, for the tetrahedral mesh type this number is more 20% since the maximum mesh flow disorientation exists in this mesh domain. Based on these observations, the use of the mixing efficiency parameter in grid convergence analysis needs to be approached cautiously. In several micromixer studies [9,27,42,43], selected mesh densities for grid independence analysis are quite close to each other which can cause misinterpretation of grid study outcome especially for high Pe situations. Namely, although grid convergence results may show small differences between mesh densities, the overall effects of numerical diffusion will remain in the simulations of selected mesh density. Therefore, based on the results reported in this study, use of a constant refinement ratio between mesh levels is recommended. Since this approach reveals the discrepancy between results in terms of numerical diffusion effects and mixing efficiency, its use needs to be avoided in GCI studies.

## 4. Results and Discussion

### 4.1. FVM Analysis

**Figure 11:**
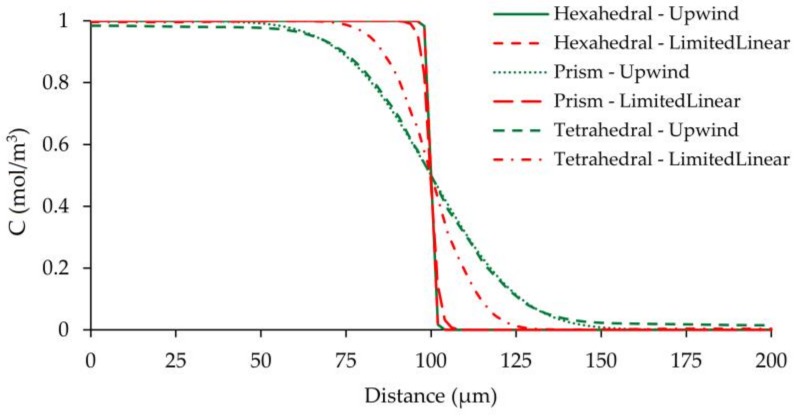
Outlet concentration distribution along the width of the mixing channel at *z* = 50 µm for all mesh types at L_1_ grid size at Re = 100 scenario.

In advection dominant transport, numerical diffusion mainly occurs due to the truncation errors of the advection term. To minimize the amount of numerical diffusion generated several high-order numerical schemes are proposed. Unfortunately, a problem-free solution that overcomes this numerical difficulty entirely is not possible. Although higher order algorithms provide more accurate solutions and may resolve higher gradients, they usually suffer from numerical instabilities which affects the reliability of the numerical solution. In three-dimensional flow systems, higher order schemes need to be used to obtain numerically less diffusive results. In FVM analysis, first-order upwind scheme and several second-order accurate numerical schemes (i.e., QUICK, MUSCL, second-order upwind, and *limitedLinear*) were tested for discretization of advection terms in the AD equation. Simulations showed that QUICK, MUSCL, and second-order upwind schemes showed oscillatory behaviors in some regions depending on the magnitude of the Reynolds number and grid size of structured prism and unstructured tetrahedral mesh types. On the other hand, although amplitudes were not high as for the other two mesh types, tested schemes also exhibited small concentration fluctuations in the hexahedral mesh simulations which varied with Re and grid size. However, stable solutions were obtained for all mesh types, grid sizes, and Re scenarios using the first-order upwind scheme and the *limitedLinear* scheme, which is a type of total variation diminishing scheme (TVD) [14]. Comparison of *limitedLinear* and first-order upwind schemes for L_1_ grid size of all mesh types at Re = 100 is given in Figure 11.

Figure 11 shows that the amount of numerical diffusion produced by first-order upwind scheme is very close to that of *limitedLinear* scheme when hexahedral mesh type is used in the computational domain. Therefore, when a good mesh and flow alignment is sustained as existed in hexahedral mesh type, the amount of numerical diffusion produced by first-order upwind scheme is insignificant. However, for other mesh groups there is a significant discrepancy between solutions of the two different algorithms since numerical diffusion effects are predominant in upwind solutions. The smearing of sharp gradient is at the maximum when prism and tetrahedral mesh types are combined with first-order upwind discretization scheme. On the other hand, while hexahedral and prism type meshes produce similar results when *limitedLinear* algorithm is employed, the tetrahedral mesh solution diverges from the sharp front. Thus, these results showed that alignment of streamlines with grid boundaries is critical in terms of numerical diffusion errors. Therefore, in the present study, the *limitedLinear* scheme was used in all simulations of FVM for consistency between all mesh types.

In FVM simulations, first stationary velocity field was obtained in the T-shape passive micromixer using the designated boundary conditions and simulation parameters given in Table 1 and Table 2. Computed stationary velocity domain was later employed to run two different steady-state scalar transport simulations. Initially, the steady-state AD equation was solved by setting the molecular diffusion constant to zero and this numerical solution is used to determine the effective diffusivity, which represents the amount of average numerical (or false) diffusion generated in the micromixer (i.e., *D_Effective_ ≈ D_Numerical_*). Using this solution, the mixing efficiency was calculated at the outlet of the micromixer using Equation (5). For convenience, mixing efficiency amount calculated is defined as “false mixing” since this mixing is created by numerical diffusion errors during the simulations. In a second simulation, the steady-state AD equation was solved using the physical molecular diffusion constant of the transported scalar (i.e., *D_M_* = 3 × 10^−10^ m^2^/s) and in this case, the computed effective diffusivity shows the collective effects of the numerical and molecular diffusion in the numerical solution (i.e., *D_Effective_ ≈ D_Numerical_ + D_Molecular_*). Therefore, a comparison of these two calculated effective diffusion constants may reveal which diffusion constant is predominant in the numerical solution.

**Figure 12:**
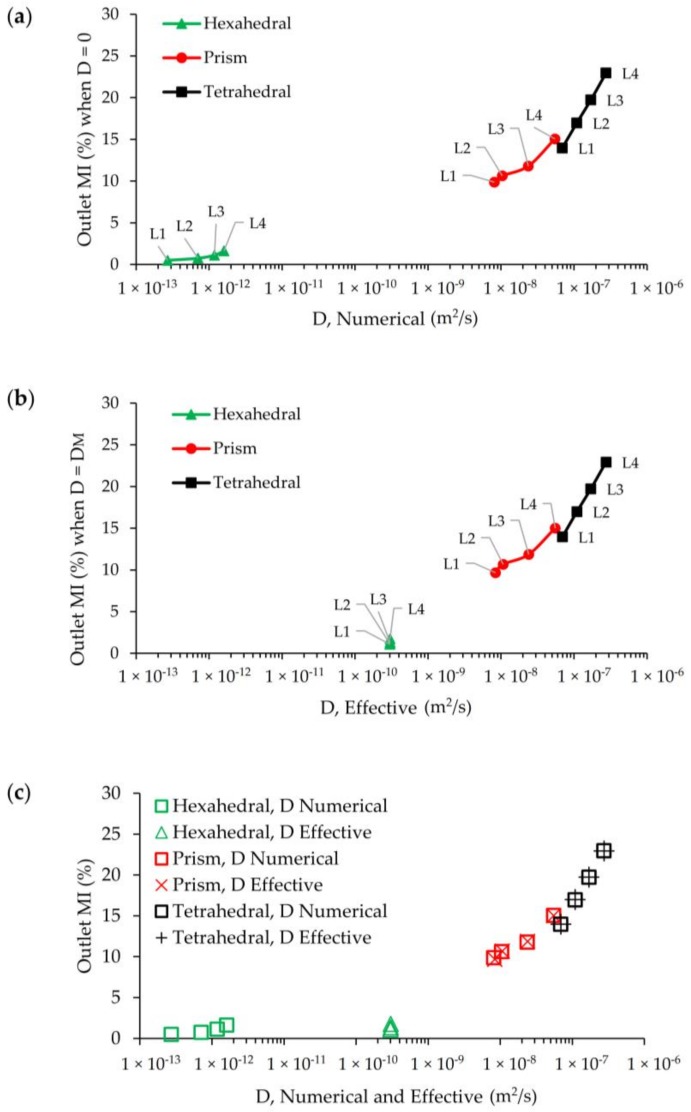
Numerical diffusion and effective diffusion in hexahedral, prism, and tetrahedral mesh domains at L_1_, L_2_, L_3_, and L_4_: (**a**) Numerical diffusion vs. MI; (**b**) effective diffusion vs. MI; (**c**). physical diffusion masking.

As it can be seen in Figure 12a, in which horizontal axis shows the average numerical diffusion error in a logarithmic scale and vertical axis shows false mixing for each mesh type and grid size at Re = 100, hexahedral mesh produced considerably lower numerical diffusion and accordingly less false mixing in contrast to prism and tetrahedral meshes. While the order of numerical diffusion is around 10^−13^ and false mixing is 0.5% at L_1_ for hexahedral mesh, these numbers sharply increased to 10^−9^ and 10% for prism and 10^−8^ and 14% for the same mesh level. Scalar transport simulations which are conducted with tetrahedral and prism mesh types produced numerical diffusion around five and four orders of magnitude higher than that of hexahedral mesh, respectively, and the magnitude of these errors manifest themselves as false mixing at the outlet. It is clear that high amount of numerical diffusion that has occurred is mainly due to non-orthogonal alignment of velocity and grid boundaries in the computational domain since other simulation parameters were all nearly constant [13]. In unstructured tetrahedral mesh type mesh flow disorientation create significant effects even at the finest mesh level in terms of false diffusion errors in the solution, but on the other side although mesh flow alignment is not maintained in prism elements, the numerical diffusion generated and false mixing obtained are noticeably lower than tetrahedral type. In prism-type mesh, a constant mesh and flow alignment is sustained through the computational domain in contrast to randomly distributed grid elements that exist in the tetrahedral type. Besides, numerical diffusion and false mixing at the outlet increases when the grid is coarser for all mesh categories. Grid coarsening effects, however show different behavior in terms of numerical diffusion and false mixing production for all mesh groups. In other words, while hexahedral mesh exhibits a minimal numerical error increase to grid size change, as it can be seen from the mild slope between mesh levels, sharply increasing slopes in the prism and tetrahedral type elements are evidence of higher rates of increase in numerical diffusion errors.

Figure 12b shows the calculated effective diffusivity and corresponding mixing efficiency at the outlet when physical diffusion is included to the solution. Calculated effective diffusivity for hexahedral mesh type is 3 × 10^−10^, implying that the physical molecular diffusion constant is completely recovered from the numerical solution of the AD equation. Thus, for all levels of hexahedral mesh, the numerical solution of the AD equation reflects the effects of the physical diffusion constant as it is much higher than the numerical diffusion magnitude shown in Figure 12a. However, for other mesh types, effective diffusivity constants and false mixing values obtained at the outlet are almost equal. This is clear when Figure 12a,b is compared. If numerical diffusion and effective diffusion constants calculated from two separate simulations and the corresponding outlet mixing efficiencies are compared, as shown in Figure 12c, it is apparent that all physical diffusion effects are completely masked by numerical diffusion effects when prism and tetrahedral mesh types are used to simulate the same scalar transport scenario. This is not the case with the hexahedral mesh type. Thus, overlapped data points from two separate simulations reveal the severity of the numerical diffusion effects. Similarly, Figure 12c reveals another important point; that is the magnitude of the calculated numerical diffusions are of several orders of magnitude lower than physical molecular diffusion even for the largest grid size of the hexahedral mesh. Therefore, considering average cell Pe numbers for different grid levels of hexahedral mesh type at Re = 100 case (given in Table 2), it is possible to obtain a solute transport solution with a negligible amount of numerical diffusion even at high average cell Pe numbers. This statement may not be correct when the flow pattern in the mixing channel turns to engulfment regime since mesh flow alignment and local velocities will change and affect the numerical diffusion that may develop.

**Figure 13:**
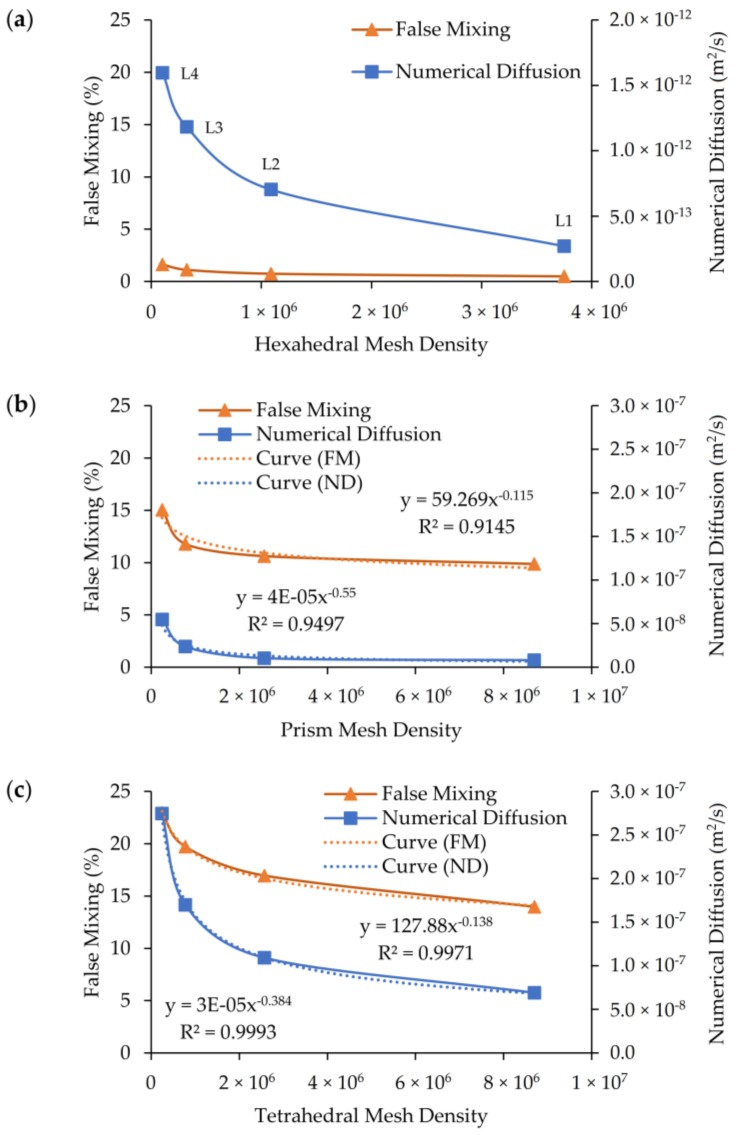
Change of numerical diffusion and false mixing with mesh density: (**a**) hexahedral; (**b**) prism; (**c**) tetrahedral.

In Figure 13, the horizontal axis shows the mesh density, the right vertical axis shows numerical diffusion outcome and the left vertical axis shows the false mixing percentage obtained in the three mesh type runs. As shown in Figure 13a–c, respectively, while false mixing is less than 2% for the coarsest grid of the hexahedral mesh (e.g., around 1 × 10^5^ cells), the false mixing is around 10% to 14% range for the prism elements and 14% to 24% range for the tetrahedral elements. We also note that for other mesh groups more than 8.5 M cells were used in the solution. When the same comparison is made for the numerical diffusion magnitudes obtained (right axis of Figure 13), one can see that while the numerical diffusion magnitude is around (10^−12^ to 10^−13^) range for hexahedral mesh, the numerical diffusion magnitudes increases to (10^−7^ to 10^−8^) range for prism and tetrahedral meshes. Also, curve fitting equations may be used to estimate the required mesh density to obtain a negligible amount of false mixing or numerical diffusion for the prism and tetrahedral element types. Table 4 shows the average mesh densities required to obtain predetermined false mixing and numerical diffusion values. To obtain a numerical diffusion equal to the molecular diffusion amount or to obtain 5% false mixing the required cell numbers are more than 10^9^ for prism and tetrahedral mesh types. These estimates are obtained from curve fitting the data in Figure 13b,c, as shown by the dotted lines identified as (Curve (FM) and Curve (ND). These estimations reveal that the required mesh densities are beyond today’s computational capacity even for the best-case scenarios in Table 4. Therefore, the results obtained confirm the previously discussed arguments that maintaining mesh flow alignment is crucial to significantly diminish numerical errors in an advection dominant transport system.

**Figure 14:**
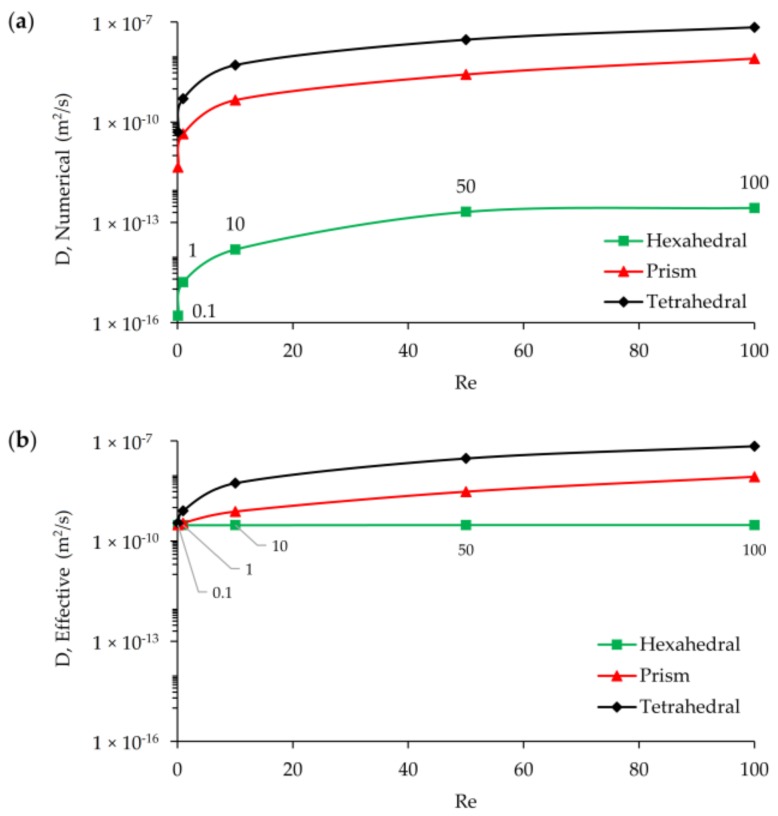
Numerical and effective diffusion change with Re number at L_1_: (**a**) numerical diffusion vs. Re; (**b**) effective diffusion vs. Re.

The relationship between Re number and numerical diffusion may also be investigated, as shown in Figure 14a. While the order of numerical diffusion is about 10^−16^ at Re = 0.1 for hexahedral mesh type, the magnitude of this error increases with increasing Re number and reaches to 10^−13^ for Re = 10 case. As discussed above and shown in Figure 14, the level of numerical diffusion error is several orders of magnitude less than the molecular diffusion constant of the solute for the hexahedral mesh. As the Reynolds number increases from Re = 0.1 to Re = 100, there is an increase in the numerical diffusion magnitude for the hexahedral mesh, but the even at Re = 100 the magnitude of numerical diffusion stays less than the magnitude of molecular diffusion constant of the solute. This shows that for all Re ranges considered in this study the mixing performance obtained represents the physical mixing in the T-shape mixer. As shown in Figure 14 the numerical error always increases with an increase of the Re. However, for prism and tetrahedral elements, the numerical error is at the level of the molecular diffusion level of the solute. Thus, in Figure 14b, it is shown that the combined increase of effective diffusivity which contributes the mixing performance now includes significant numerical mixing effects which would yield erroneous results in mixing percentages reported in the literature. As can be seen in Figure 14b, the effective diffusion constant for prism and tetrahedral meshes is at the level of (1 × 10^−7^) which is much higher than the molecular diffusion constant of the solute. Whereas, for the hexahedral element case the increase in the effective diffusion constant is insignificant, thus all mixing performances reported would represent physical mixing conditions in the T-shape mixer.

These results are also summarized in Table 5 with the fractional comparisons of numerical, effective, and physical molecular diffusion constants of all mesh types and Re scenarios that are considered in this study. For all mesh types, the concentration field can be resolved with similar accuracies only at Re = 0.1 level. In addition, prism mesh provides a solution close that of hexahedral at Re = 1. However, it should be noted that although prism and tetrahedral mesh simulations provide consistent results with hexahedral type at Re = 0.1, these mesh groups use almost 2.3 times more cells than hexahedral grid type at the finest grid level. This situation will significantly affect the computational cost. For higher Re numbers, the differences in numerical errors cannot be reconciled at the mesh levels used in this study.

**Figure 15:**
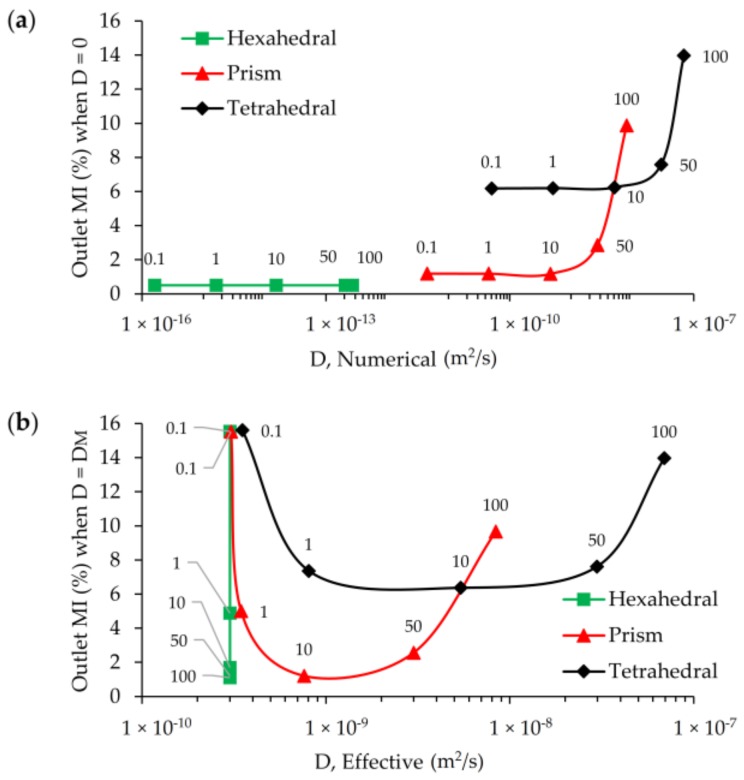
Mixing index change vs. numerical and effective diffusion for Re scenarios at L_1_: (**a**) numerical diffusion vs. MI; (**b**) effective diffusion vs. MI (inserted numbers on the data points refer to Re).

Figure 15a,b show the outlet mixing efficiencies in terms of numerical and effective diffusivities for different Re scenarios at L_1_ grid level. According to Figure 15a, although hexahedral mesh generates numerical diffusion with increasing Re number (or increasing average cell Pe number), these errors are several orders of magnitude less than physical diffusion constant. Thus, the transport solution does not produce a significant amount of false mixing at the outlet due to the maintained orthogonality between grids and flow. This situation is similar for Re = 0.1, 1, and 10 cases when prism and tetrahedral mesh types are employed for scalar transport solution. However, false mixing sharply increases after Re = 10 scenario since the effect of numerical diffusion increases sharply for high Re. At Re = 10, 50, and 100, the numerical diffusion produced for the prism element are on the order of 4.6 × 10^−10^, 2.7 × 10^−9^, and 8.0 × 10^−9^, respectively. The same trend is also observed in the tetrahedral element case yielding one order of magnitude higher numerical diffusion and much higher false mixing. For these type of meshes, a greater amount of numerical diffusion generated would lead to much higher amount of false mixing at the outlet. In Figure 15b, the effective diffusion constant and outlet mixing efficiency results are shown which highlights this point. In Figure 15b, while all three mesh types provide consistent mixing efficiencies at Re = 0.1, this is only limited to smallest Re scenario because when Re number increases the behavior of three different mesh types start diverging. At Re = 0.1 effective diffusivity represents the molecular diffusion constant of transported scalar and mixing performance of the T-mixer at the outlet is around ~16% as predicted mainly for the hexahedral mesh with the other mesh types being close to this value. When the Re increases the mean residence time of the solute in T-mixer reduces which leads less mixing efficiency in the mixer since fluid particles spend less time in the mixer. As the estimated effective diffusion constants represent physical molecular diffusion in all Re scenarios for the hexahedral mesh type, for other mesh groups, effective diffusivity increases with Re number due to increasing numerical diffusion as shown in Figure 15a. Therefore, for all Re scenarios of hexahedral mesh, the real physical diffusion constant (*DM* = 3 × 10^−10^ m^2^/s) is represented by effective diffusivity, and mixing performance decreases with increasing Re. In the case of tetrahedral mesh type, deviation from the physical molecular diffusion point (*DM* = 3 × 10^−10^ m^2^/s) starts at Re = 1 scenario and causes around 3% more mixing at the outlet than for hexahedral mesh due to the numerical diffusion effects that are generated. In this mesh type, it is observed that although the numerical diffusion is increasing for Re = 1, 10, 50 scenarios, these effects are not reflected as physical outlet mixing since there is a compensation between residence time decrease, and thus expected decrease in mixing, and numerical mixing increase and thus mixing efficiency increase. However, this compensation balance is lost after Re = 50 since numerical diffusion effects and thus false mixing significantly increases and overcomes the effects of residence time decrease. Similar trends are also observed for tetrahedral elements but in this case the false mixing effects are much higher, as can be seen in Figure 15b.

### 4.2. FEM Analysis

In FEM analysis, simulations were only performed for L_1_, L_2_, L_3_, and L_4_ grid levels at Re = 100 and for L_1_ grid level at Re = 0.1 of hexahedral mesh type using the same boundary conditions and simulation parameters with FVM. These scenarios are selected to show the effects of artificial diffusion stabilization method for advection dominant systems in FEM. When cell Pe is greater than two, the solution of scalar transport equation gives oscillatory solution in FEM depending on the magnitude of cell Pe number. In the COMSOL software, two techniques are provided to overcome instabilities during the solution of scalar transport equation that are identified as “consistent” and “inconsistent” stabilization. In consistent stabilization approach a stable solution is obtained by increasing the physical diffusion constant in the streamline direction and crosswind direction which is orthogonal to the streamlines. In the inconsistent method, the physical molecular diffusion constant is increased to reduce cell Pe number to approximately two to guarantee stability in the solution as described below.

(11) $${\mathrm{Pe}}_{\Delta }=\frac{\bar{U}\Delta x}{D_{M}}$$

(12) $${\mathrm{Pe}}_{\Delta }=\frac{\bar{U}\Delta x}{D_{M}+D_{AD}}$$

**Figure 16:**
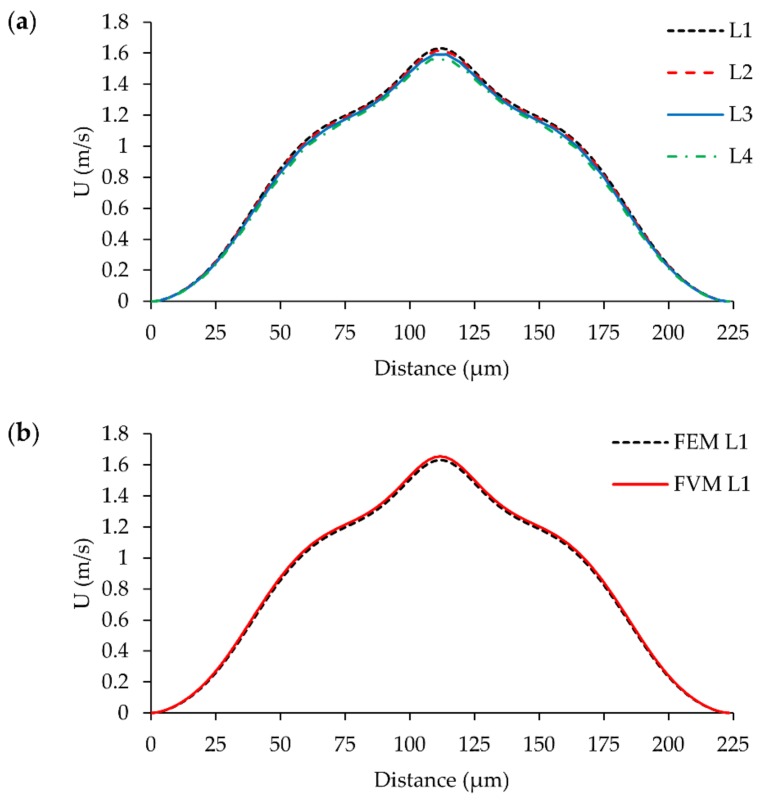
Velocity distribution on the plane at *x* = 200 µm for Re = 100 scenario: (**a**) different mesh levels of FEM; (**b**) comparison of FEM and FVM at L_1_.

Equation (11) defines average cell Pe number in which $\bar{U}$ is average velocity, ∆*x* is grid size, and *D_M_* is molecular diffusion constant. In this study Pe_∆_ is defined as average using the average velocity in the mixing channel since velocity magnitudes change at each cell in the computational domain. To decrease the average cell Pe number to a moderate number, diffusion constant is increased as described in Equation (12) in which *D_AD_* is the artificial diffusion constant. It is defined as $D_{AD}=\delta \bar{U}\Delta x$, in which δ is a tuning factor, 0 < δ < 1. In FEM simulations, this value is selected as 0.25 and 0.50 to show artificial diffusion effects in two different magnitudes. The tuning parameters applied (0.25 and 0.50) reduce the average cell Pe number to 4 and 2, respectively, for Re = 100 scenario, and 2.22 and 1.43 for Re = 0.1 case. Similarly, the FEM also requires stabilization to resolve the flow field solution; however, as mentioned earlier, high kinematic viscosity of the fluid significantly reduces the grid Re number to moderate values at which solution does not show oscillatory trend. Figure 16a shows the velocity distribution at *x* = 200 µm plane in the mixing channel at Re = 100 scenario for four different mesh levels of hexahedral type. Differences between mesh levels are close to FVM solution. Also, Figure 16b confirms that both FVM and FEM resolved the flow field with an insignificant difference.

**Figure 17:**
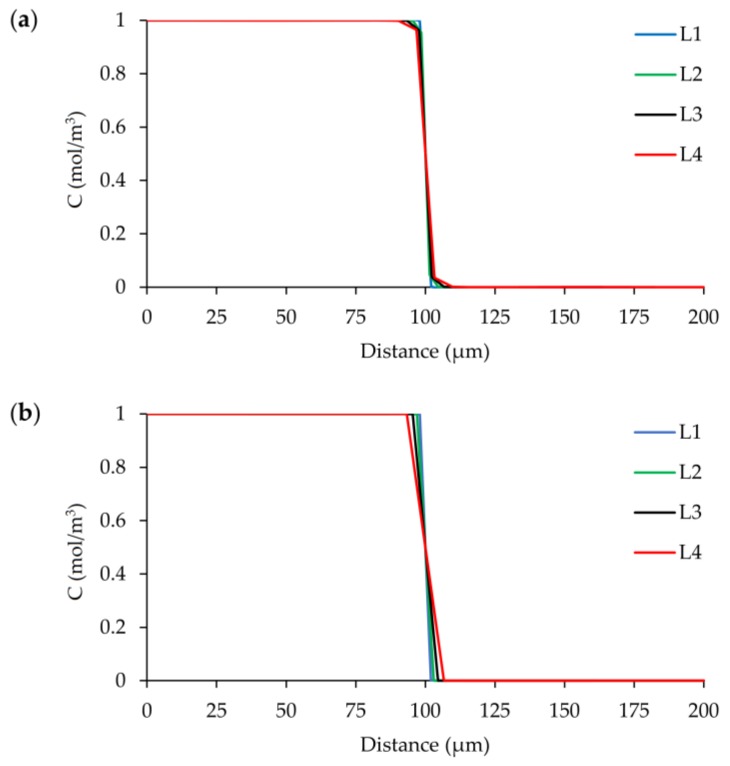
Outlet concentration distribution along the width of the mixing channel at *z* = 50 µm for four different grid levels at Re = 100 scenario: (**a**) FEM solution with consistent stabilization; (**b**) FVM solution.

Figure 17a shows concentration distribution of the Re = 100 case at the outlet of the mixer obtained from FEM solution with consistent stabilization method. Scalar transport simulations for all different mesh levels did not show an oscillatory behavior in the solution. On the other hand, while the resolved concentration field is consistent with FVM solutions as shown in Figure 17b, a small difference between the two solutions arises at L_4_ grid size. However, both methods successfully captured the sharp front in the mixing channel during the simulations. Therefore, it is obvious that consistent algorithm performs stabilization adequately.

**Figure 18:**
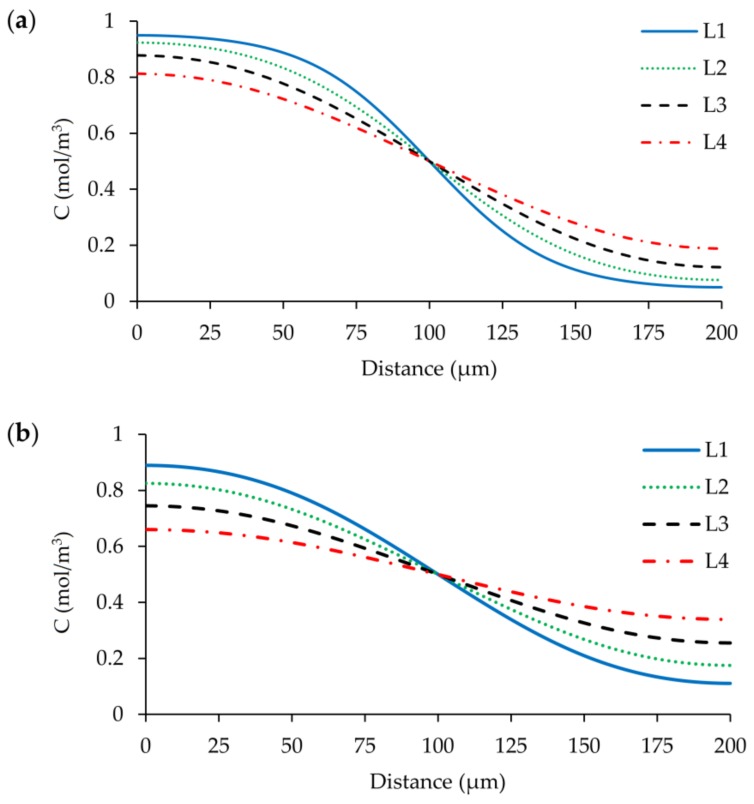
FEM solution outlet concentration distribution along the width of the mixing channel at *z* = 50 µm for four different grid levels at Re = 100 scenario: (**a**) δ = 0.25; (**b**) δ = 0.50.

When inconsistent stabilization is used in the simulations, results exhibit a considerable difference between consistent method as shown in Figure 18a,b. When tuning parameter is set to 0.25, which reduces the average cell Pe number to 4, sharp concentration profile significantly smears. This situation is worse when the tuning parameter is increased to 0.50 (average cell Pe = 2) and the concentration profiles are flattened noticeably. Also, the divergence in the solution increases with coarsening grid sizes. These are obviously the effects of artificially added diffusion amount. Although the solution of AD equation does not show any instabilities around cell Pe number = 2, the effects of artificial diffusion in the system are not tolerable in terms of a performance evaluation of mixing in the micromixer. As it can be examined from Equation (11), another possibility is decreasing grid size to reduce the average cell Pe number to 2; however, this method is not feasible since the required mesh density will be on the order of 10^10^.

### 4.3. Comparison of FVM and FEM Solutions

**Figure 19:**
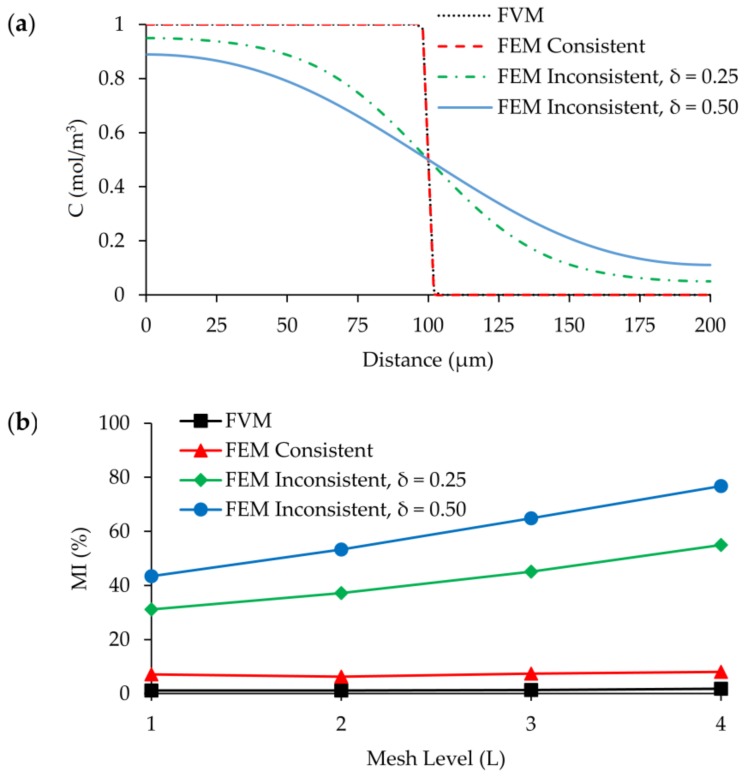
Comparison of FVM and FEM simulations at Re = 100: (**a**) Concentration distribution on the outlet cross section at *z* = 50 µm and L_1_ grid size; (**b**) MI at the outlet for four different grid levels.

When FVM and FEM are compared, a significant variance is observed between solutions in the case of an inconsistent stabilization method being used in FEM. As we plot Figure 17 and Figure 18 together in Figure 19a, an important discrepancy emerges between the results. The inconsistent stabilization cases cannot capture the actual concentration profile at the outlet of the mixing channel and provide completely discrete concentration distribution depending on the size of the tuning factor. If the performance of the micromixer is evaluated by using the artificial diffusion method, additional diffusion in the system will completely mask the real mixing efficiency which is created by the molecular diffusion constant of the solute. As it is shown in Figure 19b, there is a negligible difference between FEM with consistent stabilization and FVM, in terms of predicted outlet mixing efficiency of the micromixer. However, when the inconsistent method is used, mixing performance of T-mixer increases up to 75% at L_4_ grid size when the tuning parameter is selected as 0.50. The negative effects of the inconsistent method are still significant even for the best case (L_1_ with 0.25 regulation coefficient), in which calculated mixing efficiency is more than 30%.

**Figure 20:**
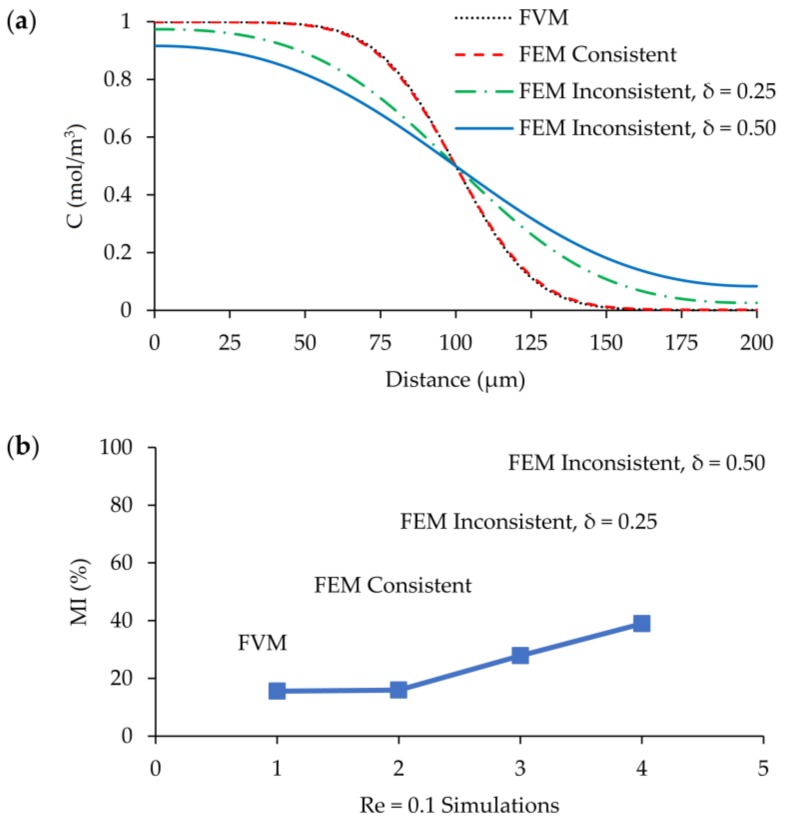
Comparison of FVM and FEM simulations at Re = 0.1 and L_1_ grid size: (**a**) concentration distribution on the outlet cross section at *z* = 50 µm; (**b**) MI at the outlet.

The same situation is also observed at the Re = 0.1 scenario of hexahedral mesh. FEM with consistent stabilization and FVM show almost the same concertation distribution at the outlet of the mixer and both methods provide equal amount of mixing at the outlet as shown in Figure 20a,b. On the other hand, although inconsistent methods capture different concentration profiles, the magnitude of smearing is decreased due to decreasing velocity magnitudes in the Re = 0.1 scenario. Nevertheless, the predicted outlet mixing efficiencies are still 2 (e.g., when δ = 0.25) and 2.5 (e.g., when δ = 0.50) times more than that of FVM and FEM with consistent solution. Therefore, considering all the outcomes discussed in this section, the micromixer performance may significantly change artificially depending on the FEM stabilization technique employed. By use of the consistent stabilization method, the results are almost uniform with FVM solutions; however, when the inconsistent correction method is employed in FEM, mixing efficiencies may increase depending on the magnitude of the tuning parameter selected. Thus, the stabilization method employed in FEM is crucial to reliably evaluate the mixing performance of a micromixer. In several numerical micromixer studies in which FEM is employed, the type of stabilization technique employed is not discussed and reported. However, based on the selected correction method, the mixing performance of the micromixer may be misevaluated and documented efficiencies may become susceptible relative to the physical mixing efficiency performance of the device.

## 5. Conclusions

In this study, mixing performance analysis of a T-shape micromixer was conducted using two frequently used CFD tools, namely FVM and FEM. The effects of different flow, transport, and mesh parameters were examined in terms of their effects on the evaluation of mixing performance of passive micromixers. In FVM, three types of mesh structure at four different grid levels (i.e., hexahedral, prism, and tetrahedral) were designed and different flow and transport scenarios were tested. The grid convergence analysis reported shows that mixing parameters should not be used as the criteria to evaluate grid convergence. This is because mixing parameters are significantly affected by numerical errors which may mislead the grid convergence analysis. It is shown that all mesh types selected were able to resolve the flow field accurately since all mesh groups captured the same flow profile at Re = 100 scenario. Meanwhile, keeping flow direction and grid boundaries aligned in the computational domain (hexahedral mesh case) produced a negligible amount of numerical diffusion in the solution of the AD equation for all Re cases tested in T-shape micromixer. On the other hand, disoriented mesh and flow direction caused severe numerical diffusion in the numerical solution of the concentration field at Re scenarios larger than 0.1. At the Re = 0.1 (average cell Pe number = 5) scenario, hexahedral, prism, and tetrahedral mesh types produced similar amounts of numerical diffusion and predicted almost equal amounts of physical diffusion and mixing at the outlet of the mixer. Therefore, numerical results showed that the false diffusion amount generated is mostly related to both cell Pe number and orthogonality between flow direction and grid lines. On the other hand, although the false diffusion generated and the physical mixing predicted were almost equal for all three mesh groups, prism and tetrahedral mesh types used 2.3 times more mesh elements than that of hexahedral at L_1_. Thus, using the hexahedral mesh type is advantageous in terms of computational cost. This is not the case for high Re and higher grid levels in which case false diffusion effects significantly increase, masking the molecular diffusion effects. Considering the outcomes, it is recommended that prism and tetrahedral grid types should be avoided, especially for solution of the AD equation. On the other hand, FEM results showed that while the consistent stabilization method provided almost the same results with FVM for the solution of both flow and transport equations, the inconsistent method, in which the physical diffusion constant is artificially increased, severely changed the calculated concentration values and artificially increased the mixing performance of the micromixer. Therefore, when FEM is used in micromixer studies, the numerical stabilization technique employed should be reported to properly evaluate the results.

## Figures and Tables

**Table 1:** Fluid properties and boundary conditions.

Simulation Type	Material Properties	Boundary	Boundary Condition
Incompressible Fluid Flow	ρ = 1000 kg/m^3^ µ = 0.001 Pa·s	Inlet 1	Uniform Inflow
		Inlet 2	Uniform Inflow
		Outlet	*p* = 0
		Walls	No-Slip
Advective-Diffusive Transport	*D* = 3 × 10^−10^ m^2^/s	Inlet 1	*c* = 0 mol/m^3^
		Inlet 2	*c* = 1 mol/m^3^
		Outlet	∂*c*/∂**n** = 0 *
		Walls	∂*c*/∂**n** = 0

* **n** is the unit vector outer normal to the wall.

**Table 2:** Test cases and simulation parameters for hexahedral, prism, and tetrahedral mesh.

**Inlet Velocity (m/s)**	**Mixing Channel**	
	**Re**	**Pe**
0.00075	0.1	3.33 × 10^2^
0.0075	1	3.33 × 10^3^
0.075	10	3.33 × 10^4^
0.375	50	1.67 × 10^5^
0.75	100	3.33 × 10^5^
**Mesh Level**	**Constant Flow, Re = 100**	
	Average Cell Re *	Average Cell Pe *
L_1_: Δ*x* = 2.0 µm	1.50	5000
L_2_: Δ*x* = 3.0 µm	2.25	7500
L_3_: Δ*x* = 4.5 µm	3.38	11250
L_4_: Δ*x* = 6.6 µm	4.95	16500
**Re**	**Constant Grid Level, L_1_**	
	**Average Cell Re**	**Average Cell Pe**
0.1	0.0015	5
1	0.015	50
10	0.15	500
50	0.75	2500
100	1.5	5000

* Average cell Re—Pe numbers are calculated for structured hexahedral mesh.

**Table 3:** Mesh properties and Grid Convergence Index (GCI) results for Re = 100 case.

**Mesh Level (L)**	**Grid Size, Δ*x* (µm)**	**Number of Cells in Computational Domain**		
		**Hexahedral**	**Prism**	**Tetrahedral**
L_1_	2.0	3.75 × 10^6^	8.70 × 10^6^	8.70 × 10^6^
L_2_	3.0	1.09 × 10^6^	2.57 × 10^6^	2.57 × 10^6^
L_3_	4.5	3.22 × 10^5^	7.72 × 10^5^	7.74 × 10^5^
L_4_	6.6	1.02 × 10^5^	2.46 × 10^5^	2.44 × 10^5^
**Mesh Level (L)**	**Grid Size, Δ*x* (µm)**	**Max Velocity at *x* = 200 µm Plane (m/s)**		
		**Hexahedral**	**Prism**	**Tetrahedral**
L_1_	2.0	1.65617	1.65691	1.65900
L_2_	3.0	1.65242	1.65175	1.65398
L_3_	4.5	1.64624	1.64361	1.64049
L_4_	6.6	1.63322	1.62657	1.61615
**Mesh Level Comparison**		**GCI Between Mesh Levels (%)**		
		**Hexahedral**	**Prism**	**Tetrahedral**
L_1_–L_2_		0.44	0.68	0.22
L_2_–L_3_		0.57	0.82	0.94
L_3_–L_4_		0.89	1.18	2.31

**Table 4:** Estimation of mesh densities in the T-mixer to reach predetermined thresholds for prism and tetrahedral mesh at Re = 100 (Pe = 3.33 × 10^5^).

False Mixing (%)	Required Average Mesh Density		Numerical Diffusion	Required Average Mesh Density	
	Prism	Tetrahedral		Prism	Tetrahedral
0.50	1.08 × 10^18^	2.81 × 10^17^	1.00 × 10^-13^	4.37 × 10^15^	1.19 × 10^22^
1.00	2.61 × 10^15^	1.85 × 10^15^	1.00 × 10^−12^	6.64 × 10^13^	2.96 × 10^19^
2.00	6.28 × 10^12^	1.22 × 10^13^	1.00 × 10^−11^	1.01 × 10^12^	7.37 × 10^16^
5.00	2.18 × 10^9^	1.59 × 10^10^	3.00 × 10^−10^	2.08 × 10^9^	1.05 × 10^13^

**Table 5:** Comparisons of numerical, effective, and physical diffusion constants in terms of different mesh type and Re scenarios at L_1_ mesh density.

Re	D-Numerical/D-Physical			D-Effective/D-Physical		
	Hexahedral	Prism	Tetrahedral	Hexahedral	Prism	Tetrahedral
0.1	5.36 × 10^−7^	0.01	0.17	1.00	1.01	1.17
1	5.39 × 10^−6^	0.15	1.69	1.00	1.15	2.69
10	5.16 × 10^−5^	1.53	17.03	1.00	2.53	18.03
50	6.91 × 10^−4^	8.99	98.32	1.00	9.99	99.32
100	9.04 × 10^−4^	26.94	230.01	1.00	27.94	231.01
